# Portable ultrasound assessment of jugular venous pressure is an accurate method for estimating volaemic status in patients with cardiac disease

**DOI:** 10.1007/s40477-022-00654-7

**Published:** 2022-03-15

**Authors:** Sam Jenkins, Patrick Knowles, Norman Briffa

**Affiliations:** 1 Department of Infection, Immunity and Cardiovascular Disease, University of Sheffield Medical School, Sheffield, S10 2RX UK; 2 Sheffield Teaching Hospitals NHSFT, Sheffield, UK

**Keywords:** Heart failure, Ultrasound, Jugular venous pressure, Central venous pressure, Diagnostic accuracy

## Abstract

**Purpose:**

The objective of this study was to determine whether ultrasound-measured jugular venous pressure (U-JVP) could accurately estimate central venous pressure (CVP).

**Methods:**

This prospective, diagnostic, single-centre study was performed at the Cardiac Intensive Care Unit of the Northern General Hospital, Sheffield, UK. Post-cardiac surgery patients were recruited from January to May 2019. The investigators were blinded to the central venous pressure when measuring the jugular venous pressure. U-JVP and direct CVP were measured simultaneously. Measurements were taken whilst the patient was ventilated and then repeated when the patient was extubated, providing non-ventilated readings.

**Results:**

One-hundred and fourteen consecutive participants with a male predominance of 71% and mean age of 65 ± 12 years were included in the analysis. Bland–Altman plots revealed that U-JVP marginally overestimated CVP by 0.91 mmHg (95% limits of agreement were −2.936 to 4.754) in ventilated patients and by 0.11 mmHg (95% limits of agreement between −2.481 and 2.695) in non-ventilated patients. Reasonable sensitivity and specificity of ultrasound-measured jugular venous pressure was achieved for low and high central venous pressure in both ventilated and non-ventilated patients.

**Conclusion:**

U-JVP accurately estimates cardiac filling pressure and fluid status in patients after cardiac surgery, irrespective of their ventilatory status. Jugular venous pressure measurement by insonation is a reliable technique that can be taught to medical students and other health professionals to non-invasively estimate central venous pressure and may be useful for assessment of volaemic status in patients with heart failure.

**Trial registration:**

ClinicalTrials.gov public (identifier NCT03815188).

**Graphical abstract:**

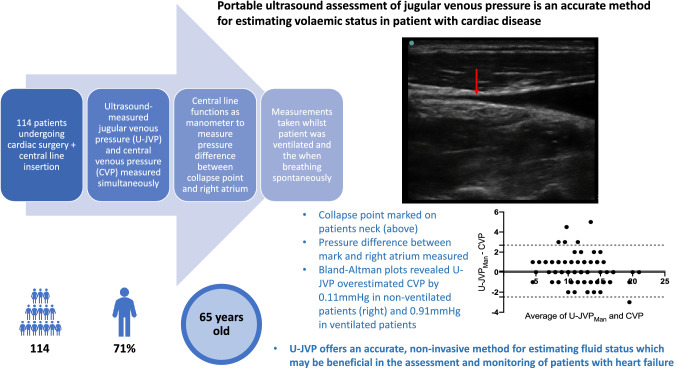

**Supplementary Information:**

The online version contains supplementary material available at 10.1007/s40477-022-00654-7.

## Introduction

Pressure and volume overload play a key role in the development of congestion and resultant clinical syndrome in heart failure (HF) [1, 2]. Symptoms and signs such as dyspnoea, orthopnoea, ankle swelling, palpitations and body weight are all markers of HF [3]. These markers usually present late, meaning that early detection and treatment is difficult, which may ultimately impact on the morbidity and mortality of patients with HF [4]. Non-invasive methods which can accurately measure baseline and ongoing changes in fluid status may enable earlier detection of HF or earlier step-up in management in those with worsening disease.

Central venous pressure (CVP) is the blood pressure in the large central veins which drives blood from the body to the right side of the heart. As they arise directly from the right atrium with no intervening valves, pressure in these veins reflects the pressure in the right atrium. Measurement of CVP therefore allows for estimation of fluid status and filling pressures of the heart. CVP is directly measured with a central venous catheter in patients who are severely ill including septic and burn patients, as well as those undergoing major surgery [5]. Estimation of the jugular venous pressure (JVP) in the neck is the classic method to predict fluid status on physical examination that is taught early in all medical schools for assessment of central venous pressure [6]. As a physical sign, however, it is notoriously difficult to get right, and doubts about its validity have always existed. Increasingly many medical students are unable to convincingly demonstrate an elevated JVP or how to express the result in centimetres of water because they have not been taught to.

Lipton [7] first described the technique of using ultrasound to visualise the top of the blood column in the internal jugular vein (IJV). This provides an alternative method for accurately measuring JVP compared to clinical examination. Studies designed to validate the use of ultrasound to measure JVP (U-JVP) have reported inconsistent findings [8–11]. We hypothesised that by addressing the potential limitations of these studies, we could demonstrate that U-JVP accurately reflects the CVP in both ventilated patients and those breathing spontaneously.

## Methods

### Location, patients and study design

**Fig. 1:**
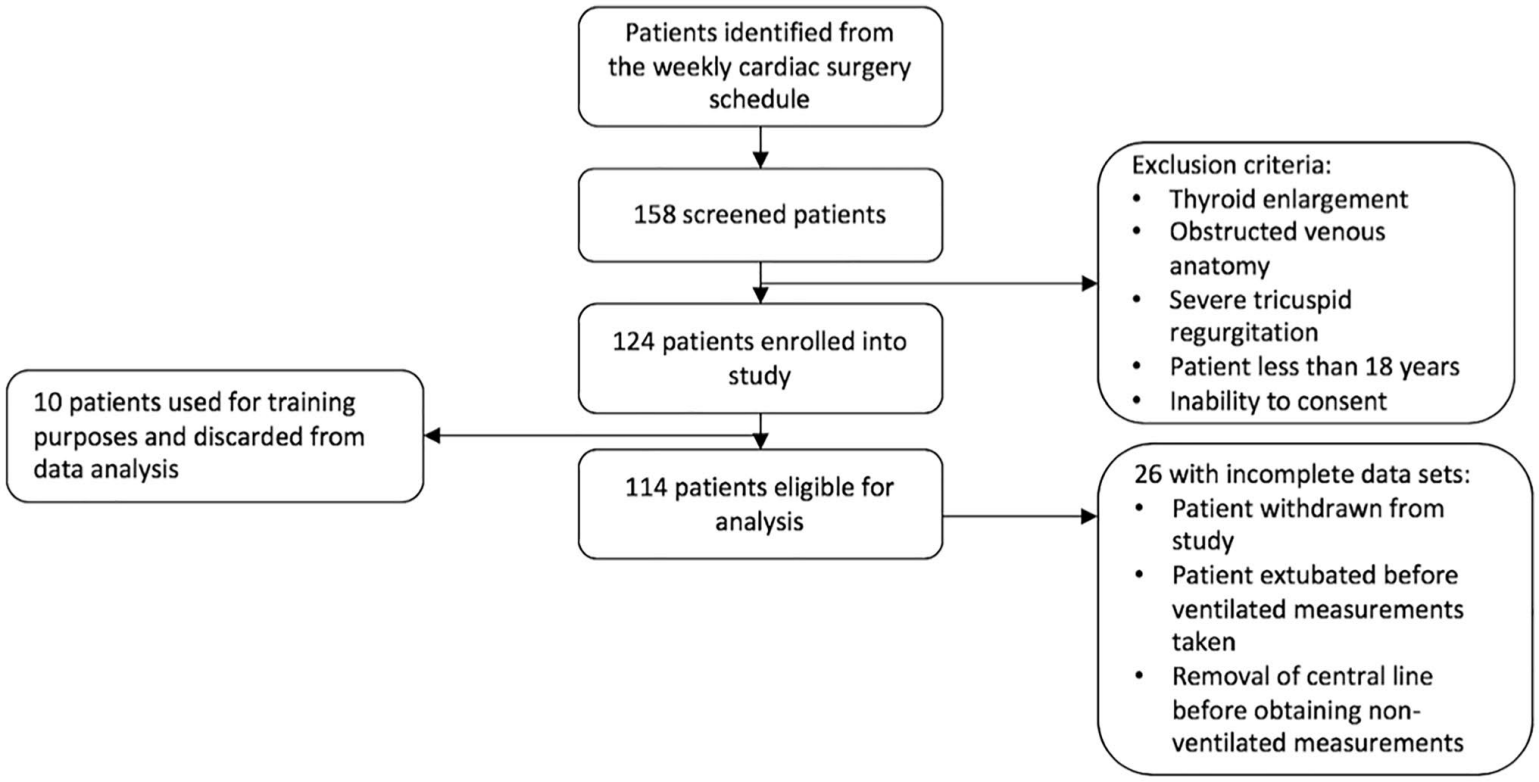
Study design flowchart

We performed this prospective study in the Cardiac Intensive Care Unit of the Northern General Hospital, Sheffield, UK between January and May 2019. Study subjects consisted of patients undergoing open heart surgery requiring insertion of a central venous catheter. They were identified from the weekly cardiac surgery schedule and recruited consecutively (Fig. 1). Patients with an enlarged thyroid, with severe tricuspid regurgitation, who were under the age of 18 years or who were unable to consent were excluded from the trial. The investigation conforms with the principles outlined in the Declaration of Helsinki. Written consent was obtained from all patients, including permission to use their ultrasound images and electrical traces in this report. Approval was granted by the Research Ethics Committee and Health Research Authority. This trial was registered on the ClinicalTrials.gov public database (NCT03815188).

Post-operatively, U-JVP and the gold-standard directly measured CVP were recorded simultaneously in each patient. These measurements were taken whilst the patient was ventilated and then repeated after extubation when the patient was breathing spontaneously by one of two investigators (SJ or PK).

### Measurement techniques

**Fig. 2.**
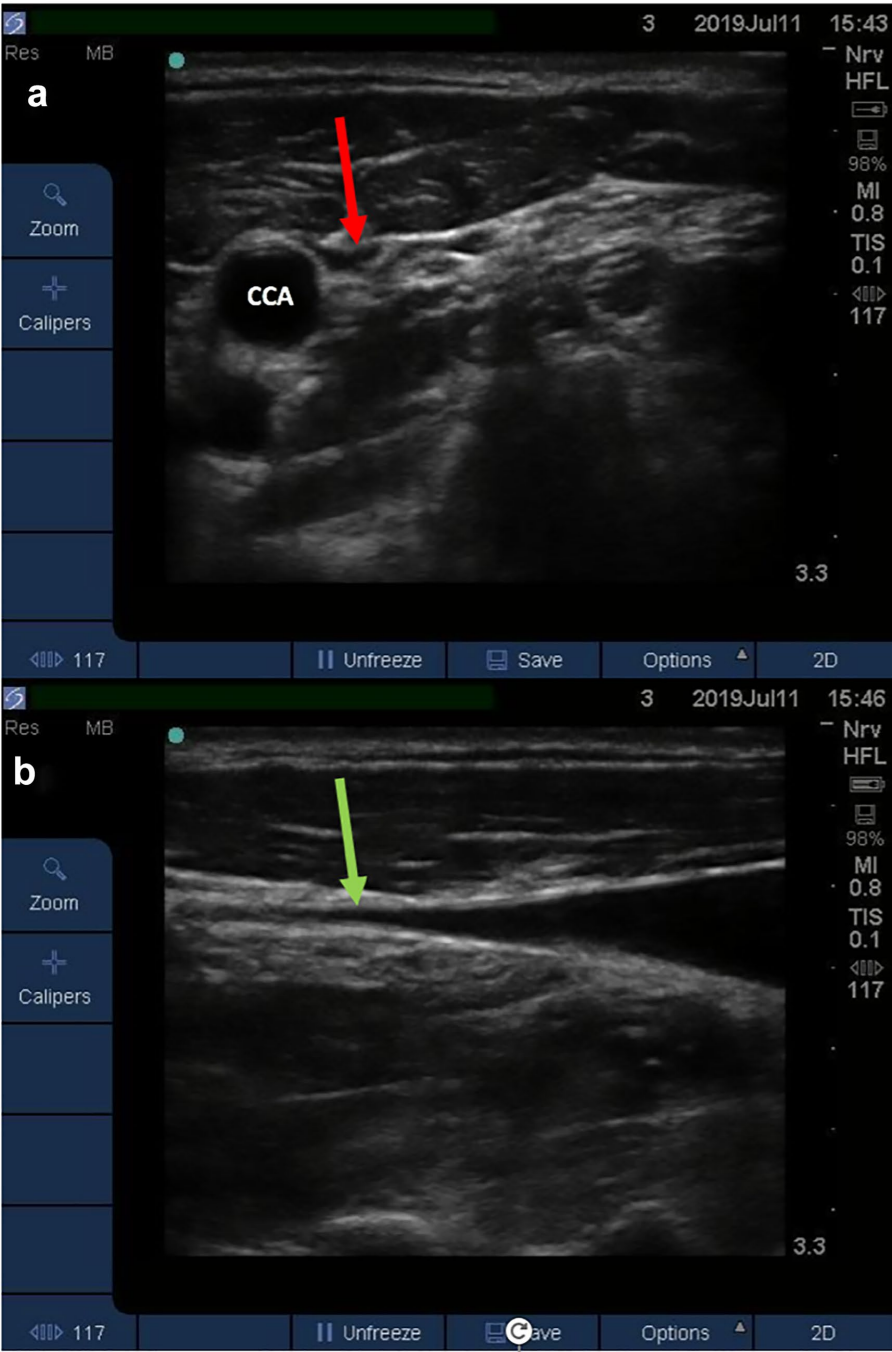
**a** Transverse view of a collapsed IJV. The red arrow points towards the collapsed IJV. The higher blood pressure within the CCA means the vessel does not collapse. **b** Longitudinal axis of the IJV. The IJV tapers until reaching the point of collapse. The green arrow indicates the point at which the vein was considered collapsed

Prior to measuring both U-JVP and CVP post-operatively, the central line transducer was placed at the phlebostatic axis in line with the patient’s right atrium. The central line was calibrated by zeroing the transducer relative to atmospheric pressure. The line was flushed to ensure there was a continuous column of fluid. For blinding purposes, estimation of the U-JVP was carried out first prior to invasive CVP measurement. A Sonosite NanoMaxx 13 MHz linear transducer or Sonosite S-Nerve 13 MHz linear transducer was used for insonation. The probe was placed on the patient’s neck in the transverse plane, proximal to the clavicle on the contralateral side to the inserted central line. The IJV was located whilst applying minimal pressure to the skin. The probe was moved superiorly until the vein was seen to collapse (Fig. 2a). The angle of the bed on which the patient was lying was adjusted to allow visualisation of the collapse point in the middle of the patient’s neck. This ensured that the vein was not being splinted open by negative intrathoracic pressure as it entered the thoracic cavity. The probe was then rotated into the longitudinal axis to identify the point of venous collapse upon end-expiration (Fig. 2b). The venous collapse point was then marked. The investigator then observed the directly measured CVP on the monitor and recorded the value immediately.

Two different techniques were then employed to measure the U-JVP: firstly, the traditional two-ruler technique and secondly using a manometer. The bed was maintained at a constant angle and the patient remained still whilst all measurements were being taken. Two rulers were placed perpendicularly and levelled to measure the vertical distance from the marked point of venous collapse to a surface point marking the position of the right atrium (U-JVP_Ruler_). The distance was converted into millimetres of mercury (1.36 cmH_2_O = 1 mmHg). In the second method, the transducer was zeroed and placed at the level of the right atrium whilst the line was detached from the patient, flushed and placed at the marked point of collapse. This measured the pressure difference between the collapse point and transducer (U-JVP_Man_). This method removed any human error which the two-ruler technique incurs and acted to validate the accuracy of U-JVP.

### Statistical analysis

A power analysis based on similar work published previously suggested a minimum sample size of 45 [8–11]. Scatter plots to determine correlation were constructed using GraphPad Prism version 8.4.3 statistical software. Pearson coefficients were used to determine any significant correlation between the two techniques. Bland–Altman plots were constructed to measure the mean difference (agreement) between techniques. The limits of agreement were then calculated. The diagnostic ability of U-JVP to estimate CVP was calculated from contingency tables. Receiver operating characteristic (ROC) curves were constructed and the area under the curve (AUC) quantified to evaluate the diagnostic sensitivity and specificity of U-JVP_Man_. A value of > 10 mmHg was considered high CVP and a value of < 6 mmHg as low CVP [8]. Missing data sets were not incorporated into subgroup analyses.

## Results

The jugular vein and collapse point were easily visualised in all patients. No adverse events were reported throughout the duration of the study.

### Patient demographics

A total of 124 patients were recruited into this study. The first 10 patients were used for training purposes and not incorporated into the analysis. Demographic data for the 114 patients recruited to the study are shown in Table 1.

**Table 1:** Demographic data for 114 patients recruited and included in the study analysis

Characteristic	Value
Age (years)	65.2 ± 11.5
Gender (*n*)	
Male	81
Female	33
Height (m)	1.7 ± 0.09
Weight (kg)	84.7 ± 18.5
BMI (kg/m^2^)	29.2 ± 5.4
Type of surgery (*n*)	
Aortic dissection repair	1
Aortic root replacement ± AVR	6
AVR ± MVR or TV repair	25
CABG ± AVR or MVS	51
Left atrial myxoma	1
Left atrial mass	1
MVS ± TVS	25
Pericardiectomy	1
Replacement of ascending aorta	3
Right IJV catheter (*n*)	113
PEEP value—ventilated patients only (cmH_2_O)	5.4 ± 0.9

*BMI* body mass index, *AVR* aortic valve replacement, *MVR* mitral valve replacement, *TV* tricuspid valve, *CABG* coronary artery bypass graft, *MVS* mitral valve surgery, *TVS* tricuspid valve surgery, *PEEP* positive end-expiratory pressure

### Correlation between methods

**Fig. 3:**
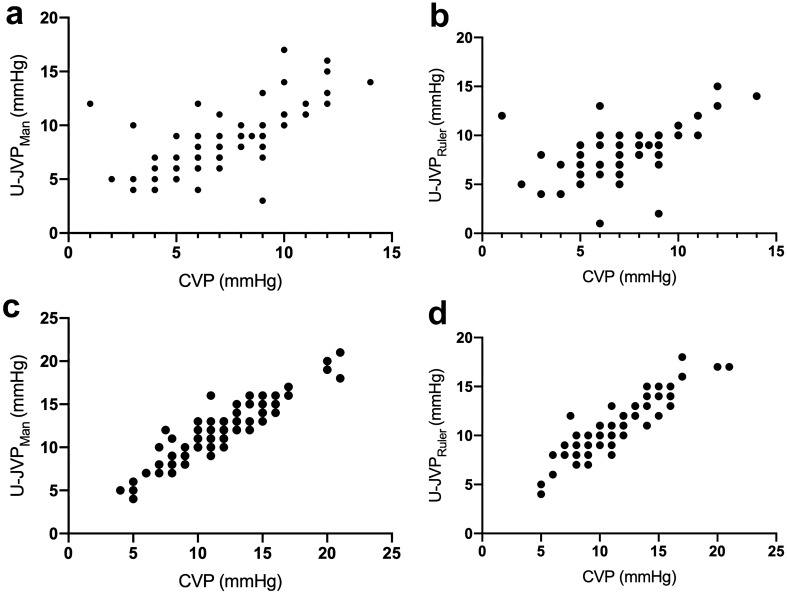
Scatter plots showing correlation between: **a** CVP and U-JVP_Man_ in ventilated patients; **b** CVP and U-JVP_Ruler_ in ventilated patients; **c** CVP and U-JVP_Man_ in non-ventilated patients; **d** CVP and U-JVP_Ruler_ in non-ventilated patients

U-JVP_Man_ showed a strong correlation with CVP in ventilated patients (*r* = 0.72, 95% CI 0.6059–0.7978, *p* < 0.0001) (Fig. 3a). The U-JVP_Ruler_ technique was only able to produce a moderate correlation with CVP, although their relationship remained highly significant (*r* = 0.63, 95% CI 0.6059–0.7978, *p* < 0.0001) (Fig. 3b). A total of 15/104 ventilated U-JVP_Man_ readings overestimated the CVP by ≥ 3 mmHg. Only a single case under-read the CVP by a pressure of 6 mmHg. The remaining 14 cases overestimated the CVP, with a mean pressure of 4.7 mmHg higher than the CVP.

U-JVP_Man_ and CVP displayed a positive correlation in spontaneously breathing patients (*r* = 0.93, 95% CI 0.8964–0.9523, *p* < 0.0001) (Fig. 3c). U-JVP_Ruler_ and CVP demonstrated almost as strong a relationship, recording a correlation coefficient of 0.90 (95% CI 0.8526–0.9392, *p* < 0.0001) (Fig. 3d). Only 6/98 patients measured a difference of ≥ 3 mmHg between the U-JVP_Man_ and CVP (one underestimation and five overestimation). In spontaneously breathing patients, overestimation of CVP by U-CVP occurred in only three patients.

### Agreement between methods

**Fig. 4:**
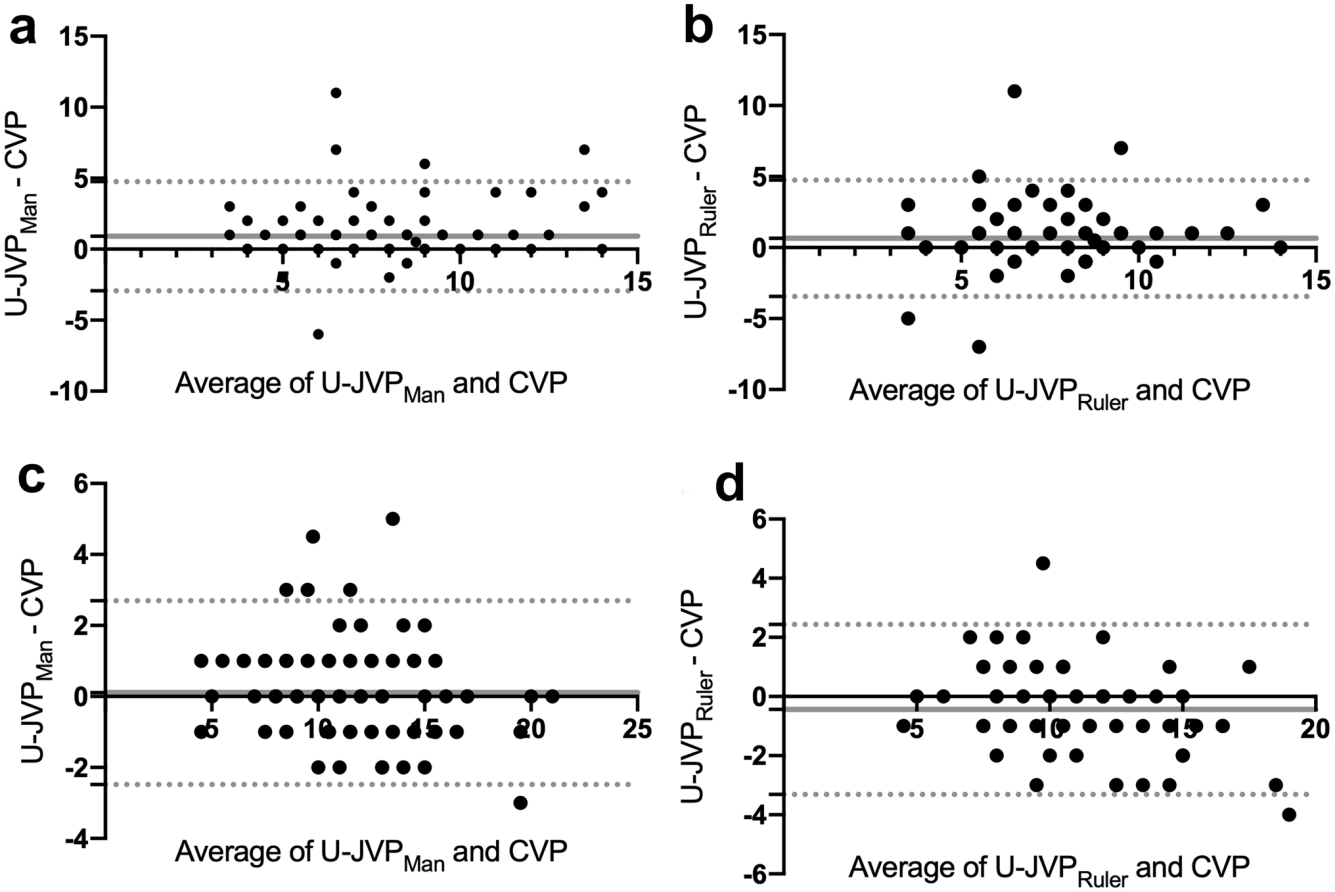
Bland–Altman plots measuring agreement between: **a** CVP and U-JVP_Man_ in ventilated patients; **b** CVP and U-JVP_Ruler_ in ventilated patients; **c** CVP and U-JVP_Man_ in non-ventilated patients; **d** CVP and U-JVP_Ruler_ in non-ventilated patients. Units are measured in millimetres of mercury. The solid grey line signifies the mean difference. The grey dotted lines indicate the 95% limits of agreement

The mean difference between U-JVP_Man_ and CVP in ventilated patients was 0.9087 mmHg (Fig. 4a). The 95% limits of agreement were −2.936 to 4.754. The mean difference between U-JVP_Ruler_ was smaller (0.6494 mmHg; 95% limits of agreement −3.446 to 4.745) (Fig. 4b).

In patients who were breathing spontaneously, differences were smaller. U-JVP_Man_ and CVP showed excellent agreement in non-ventilated patients, with a mean difference of 0.1071 mmHg and limits of agreement between −2.481 and 2.695 (Fig. 4c). A mean difference of −0.4392 mmHg and limits of agreement between −3.313 and 2.435 was measured between U-JVP_Ruler_ and CVP (Fig. 4d).

### Assessing the diagnostic ability of U-JVP

A summary of the diagnostic performance of U-JVP_Man_, including corresponding contingency tables and ROC curves, is detailed in the Supplemental Appendices (eTables 1–3, eFigures 1–3). Performing U-JVP_Man_ to estimate high CVP (> 10 mmHg) in ventilated patients produced a sensitivity of 100% and specificity of 90%. These values produced an AUC of 0.950. The test produced a positive predictive value (PPV) of 44% in contrast to a negative predictive value (NPV) of 100%. Four of the 10 patients who would have been falsely diagnosed with a high CVP of 11 mmHg arose from U-JVP_Man_ overestimating the CVP by only 1 mmHg. U-JVP_Man_ was only able to demonstrate a sensitivity of 59% from a small sample of 22 patients with low CVP readings. However in 4/9 of these patients who were falsely diagnosed with a normal CVP, U-JVP_Man_ over-read by only 1 mmHg. Therefore the CVP was estimated to be normal (6 mmHg) when the actual CVP value was 5 mmHg. This minimal discrepancy had a significant impact on the resultant sensitivity. The specificity, however, was excellent (99%), leading to an AUC value of 0.790. The PPV and NPV calculated were 93% and 90%, respectively.

A sensitivity of 92% and specificity of 82% were observed when estimating elevated CVP in spontaneously breathing patients. In three of the five patients that provided a false-negative result, U-JVP_Man_ only underestimated the CVP by 1 mmHg. The area under the ROC curve was 0.870. The PPV (89%) and NPV (86%) were also high. U-JVP_Man_ provided a false-positive elevated CVP result following an over-reading of only 1 mmHg in 3/7 cases. Only four participants recorded a low CVP, meaning the sensitivity (75%) represented a very small sample of patients. The specificity was 100% in 94 patients with a low CVP. The ROC curve had an area of 0.875. The PPV and NPV measured 100% and 99%, respectively.

## Discussion

Visual assessment of a patient’s JVP has been taught to medical students as part of a routine clinical examination since the early part of the twentieth century. It is undoubtedly useful and easy to demonstrate in conditions such as severe tricuspid regurgitation and constrictive pericarditis (Kussmaul’s sign). The more common assessment of volaemic status in patients with chronic heart failure is harder. Clinical assessment of JVP has been demonstrated to be inaccurate [6]. In addition, the high body mass index (BMI) and neck girth of patients also contributes to the increasing inability of teachers to effectively demonstrate and new students to learn the technique. In this study we have demonstrated conclusively that using ultrasound to assess CVP/JVP was feasible and accurate. It has been suggested that the IJV has an inherent mechanism of exerting venous tone which causes the JVP to underestimate CVP [9]. However, the concordant pressures measured by the JVP and CVP in our study support the theory that the IJV acts as a passive tube responding to changes in right atrial pressure (RAP) and volume.

Assessment of a patient’s fluid status is critically important in patients with heart failure [12]. A reliable clinical test that is able to non-invasively determine a patient’s volaemic status has not been described until now.

### Clinical context

The scope for use of U-JVP in the assessment, diagnosis and management of heart failure is clear to see. Our findings validate its use as an accurate measure of fluid status. Point-of-care ultrasound machines are now very portable, and this feasible technique can be employed by specialist HF nurses and other members of the heart failure team, both in hospital and in the community [13, 14]. This non-invasive and relatively inexpensive technique can be added to the armamentarium of modern techniques such as pulmonary pressure assessment (CardioMEMS) or the demonstration of intrapulmonary septal fluid with ultrasound that can be used for assessment of heart failure patients and as an accurate guide for fluid management and diuretic therapy [15].

### Limitations

Patients having undergone cardiac surgery were pharmacologically optimised in intensive care during the time of the study. Their surgery should have also corrected any abnormal JVP value that they would have had prior to their surgery. This population therefore may not entirely reflect the patient population that will benefit most from the examination. There were small sample sizes for calculating the diagnostic ability of U-JVP in some subgroups of patients. These subgroups included ventilated patients with a high CVP and non-ventilated patients with a low CVP. The accuracy of U-JVP at these ranges of CVP may require further investigation. CVP is measured on a continuous scale, and therefore U-JVP may appear to be less accurate than in reality when CVP is categorised into low or high subgroups. Awareness that U-JVP overestimates CVP by approximately 1 mmHg may be a more appropriate measure of accuracy. Additional work to identify inter-dependent variability may be warranted.

### Future application

The logical next step in the assessment of this technology is to compare it to recognised ways of fluid assessment (such as body weight or CardioMEMS) in a population of ambulant patients with heart failure using relevant questionnaires (such as the Minnesota Living with Heart Failure questionnaire) and readmissions to hospital as important outcomes. The miniaturisation of devices and the advent of handheld cardiac ultrasound means that U-JVP could play a significant role in the simple, non-invasive bedside and community assessment and monitoring of heart failure patients.

### Conclusion

In the most comprehensive study of its kind to date, we have demonstrated that U-JVP provides significant potential for diagnosing and monitoring cardiac filling pressure and haemodynamic status in both ventilated and non-ventilated patients with cardiac disease undergoing corrective surgery. Further studies will inform us of the suitability of this technique in the management of a larger population of patients with heart failure.

## Supplementary Information

Below is the link to the electronic supplementary material.

Supplementary file1 (DOCX 99 kb)

## Declarations

### Data availability

The datasets generated and analysed during the current study are available from the corresponding author on reasonable request.

## References

[1] Ponikowski P, Voors AA, Anker SD (2016). 2016 ESC Guidelines for the diagnosis and treatment of acute and chronic heart failure: the Task Force for the diagnosis and treatment of acute and chronic heart failure of the European Society of Cardiology (ESC) Developed with the special contribution of the Heart Failure Association (HFA) of the ESC. Eur Heart J.

[2] Harjola V-P, Mullens W, Banaszewski M (2017). Organ dysfunction, injury and failure in acute heart failure: from pathophysiology to diagnosis and management. A review on behalf of the Acute Heart Failure Committee of the Heart Failure Association (HFA) of the European Society of Cardiology (ESC). Eur J Heart Fail.

[3] Gheorghiade M, Follath F, Ponikowski P (2010). Assessing and grading congestion in acute heart failure: a scientific statement from the acute heart failure committee of the heart failure association of the European Society of Cardiology and endorsed by the European Society of Intensive Care Medicine. Eur J Heart Fail.

[4] Abraham WT, Adamson PB, Bourge RC (2011). Wireless pulmonary artery haemodynamic monitoring in chronic heart failure: a randomised controlled trial. Lancet.

[5] Dalrymple P (2006). Central venous pressure monitoring. Anaesth Intensive Care Med.

[6] McGee SR (1998). Physical examination of venous pressure: a critical review. Am Heart J.

[7] Lipton B (2000). Estimation of central venous pressure by ultrasound of the internal jugular vein. Am J Emerg Med.

[8] Avcil M, Kapci M, Dagli B (2015). Comparison of ultrasound-based methods of jugular vein and inferior vena cava for estimating central venous pressure. Int J Clin Exp Med.

[9] Deol GR, Collett N, Ashby A, Schmidt GA (2011). Ultrasound accurately reflects the jugular venous examination but underestimates central venous pressure. Chest.

[10] Nik Muhamad NA, Safferi RS, Robertson CE (2015). Internal jugular vein height and inferior vena cava diameter measurement using ultrasound to determine central venous pressure: a correlation study. Med J Malays.

[11] Siva B, Hunt A, Boudville N (2012). The sensitivity and specificity of ultrasound estimation of central venous pressure using the internal jugular vein. J Crit Care.

[12] Szymczyk T, Sauzet O, Paluszkiewicz LJ (2020). Non-invasive assessment of central venous pressure in heart failure: a systematic prospective comparison of echocardiography and Swan-Ganz catheter. Int J Cardiovasc Imaging.

[13] Gundersen GH, Norekval TM, Haug HH (2016). Adding point of care ultrasound to assess volume status in heart failure patients in a nurse-led outpatient clinic. A randomised study. Heart.

[14] Gustafsson M, Alehagen U, Johansson P (2015). Pocket-sized ultrasound examination of fluid imbalance in patients with heart failure: a pilot and feasibility study of heart failure nurses without prior experience of ultrasonography. Eur J Cardiovasc Nurs.

[15] Pour-Ghaz I, Hana D, Raja J (2019). CardioMEMS: where we are and where can we go?. Ann Transl Med.

