# Supplementary material for: Portable ultrasound assessment of jugular venous pressure is an accurate method for estimating volaemic status in patients with cardiac disease
Source: J Ultrasound. 2022 Mar 15;25(4):939–47. doi: 10.1007/s40477-022-00654-7 (PMC9705668; doi:10.1007/s40477-022-00654-7)
Supplement: Supplementary file 1 — Supplementary file1 (DOCX 99 kb) [file 40477_2022_654_MOESM1_ESM.docx]

**Supplementary Material**

**eTable 1 –** Summary of the diagnostic ability of U-JVP_Man_ to predict low, normal and elevated CVP values.

**eTable 2 –** Contingency tables classifying the ability of U-JVP_Man_ to predict CVP in ventilated patients.

**eTable 3 –** Contingency tables classifying the ability of U-JVP_Man_ to predict CVP in non-ventilated patients.

**eFigure 1** – ROC curves illustrating the diagnostic ability of U-JVP_Man_ to estimate CVP in ventilated patients with: 1a) elevated CVP; 1b) normal CVP; 1c) low CVP.

**eFigure 2** – ROC curves displaying the diagnostic ability of U-JVP_Man_ to estimate CVP in non-ventilated patients with: 2a) elevated CVP; 2b) normal CVP; 2c) low CVP.

**eFigure 3** – Bland-Altman plots comparing U-JVP_Man_ and CVP in ventilated patients 3a) undergoing concurrent inotrope administration; 3b) free from inotropes.

**Tables**

eTable 1 - Summary of the diagnostic ability of U-JVP_Man_ to predict low, normal and elevated CVP values.

| **Form of Ventilation** | **CVP Value** | **Total Measured** | **Total Positive** | **Sensitivity (%)** | **Specificity (%)** | **PPV (%)** | **NPV (%)** | **AUC** |
| --- | --- | --- | --- | --- | --- | --- | --- | --- |
| **Combined Ventilated + Non-Ventilated** | Elevated CVP | 202 | 63 | 93 | 87 | 79 | 96 | 0.9 |
|  | Normal CVP | 202 | 87 | 82 | 85 | 86 | 83 | 0.835 |
|  | Low CVP | 202 | 16 | 62 | 99 | 94 | 95 | 0.805 |
| **Ventilated** | Elevated CVP | 104 | 8 | 100 | 90 | 44 | 100 | 0.95 |
|  | Normal CVP | 104 | 60 | 83 | 75 | 88 | 67 | 0.79 |
|  | Low CVP | 104 | 13 | 59 | 99 | 93 | 90 | 0.79 |
| **Non-Ventilated** | Elevated CVP | 98 | 55 | 92 | 82 | 89 | 86 | 0.87 |
|  | Normal CVP | 98 | 27 | 79 | 91 | 82 | 89 | 0.85 |
|  | Low CVP | 98 | 3 | 75 | 100 | 100 | 99 | 0.875 |

**eTable 2** – Contingency tables classifying the ability of U-JVP_Man_ to predict CVP in ventilated patients.

| **Elevated CVP (>10mmHg)** |  |  | **CVP > 10mmHg** | |
| --- | --- | --- | --- | --- |
|  |  |  | Yes | No |
|  | **U-JVP_Man_**  **> 10mmHg** | Yes | 8 | 10 |
|  |  | No | 0 | 86 |
| **Normal CVP (6-10mmHg)** |  |  | **CVP 6mmHg - 10mmHg** | |
|  |  |  | Yes | No |
|  | **U-JVP_Man_**  **6mmHg - 10mmHg** | Yes | 60 | 8 |
|  |  | No | 12 | 24 |
| **Low CVP (<6mmHg)** |  |  | **CVP < 6mmHg** | |
|  |  |  | Yes | No |
|  | **U-JVP_Man_**  **< 6mmHg** | Yes | 13 | 1 |
|  |  | No | 9 | 81 |

**eTable 3** – Contingency tables classifying the ability of U-JVP_Man_ to predict CVP in non-ventilated patients.

| **Elevated CVP (>10mmHg)** |  |  | **CVP > 10mmHg** | |
| --- | --- | --- | --- | --- |
|  |  |  | Yes | No |
|  | **U-JVP_Man_**  **> 10mmHg** | Yes | 55 | 7 |
|  |  | No | 5 | 31 |
| **Normal CVP**  **(6-10mmHg)** |  |  | **CVP 6mmHg - 10mmHg** | |
|  |  |  | Yes | No |
|  | **U-JVP_Man_**  **6mmHg - 10mmHg** | Yes | 27 | 6 |
|  |  | No | 7 | 58 |
| **Low CVP (<6mmHg)** |  |  | **CVP < 6mmHg** | |
|  |  |  | Yes | No |
|  | **U-JVP_Man_**  **< 6mmHg** | Yes | 3 | 0 |
|  |  | No | 1 | 94 |

**Figures**

**eFigure 1** – ROC curves illustrating the diagnostic ability of U-JVP_Man_ to estimate CVP in ventilated patients with: 1a) elevated CVP; 1b) normal CVP; 1c) low CVP.

**1a)**

**1b)**

**1c)4**

**eFigure 2** – ROC curves displaying the diagnostic ability of U-JVP_Man_ to estimate CVP in non-ventilated patients with: 2a) elevated CVP; 2b) normal CVP; 2c) low CVP.

**2a)**

**2b)**

**2c)**

**eFigure 3** – Bland-Altman plots comparing U-JVP_Man_ and CVP in ventilated patients 3a) undergoing concurrent inotrope administration; 3b) free from inotropes.

**3b)**

**3a)**
